# Maternal Health Factors as Risks for Postnatal Depression: A Prospective Longitudinal Study

**DOI:** 10.1371/journal.pone.0147246

**Published:** 2016-01-19

**Authors:** Catherine L. Chojenta, Jayne C. Lucke, Peta M. Forder, Deborah J. Loxton

**Affiliations:** 1 Research Centre for Generational Health and Ageing, University of Newcastle, Callaghan, Australia; 2 Australian Research Centre in Sex, Health and Society, Faculty of Health Sciences, La Trobe University, Melbourne, Australia; University of Rochester, UNITED STATES

## Abstract

**Purpose:**

While previous studies have identified a range of potential risk factors for postnatal depression (PND), none have examined a comprehensive set of risk factors at a population-level using data collected prospectively. The aim of this study was to explore the relationship between a range of factors and PND and to construct a model of the predictors of PND.

**Methods:**

Data came from 5219 women who completed Survey 5 of the Australian Longitudinal Study on Women’s Health in 2009 and reported giving birth to a child.

**Results:**

Over 15% of women reported experiencing PND with at least one of their children. The strongest positive associations were for postnatal anxiety (OR = 13.79,95%CI = 10.48,18.13) and antenatal depression (OR = 9.23,95%CI = 6.10,13.97). Positive associations were also found for history of depression and PND, low SF-36 Mental Health Index, emotional distress during labour, and breastfeeding for less than six months.

**Conclusions:**

Results indicate that understanding a woman’s mental health history plays an important role in the detection of those who are most vulnerable to PND. Treatment and management of depression and anxiety earlier in life and during pregnancy may have a positive impact on the incidence of PND.

## Introduction

Postnatal depression (PND) is estimated to affect around 13% of US mothers [1], and between 10–20% of Australian mothers [2]. While several important initiatives have highlighted the need for screening and treatment of perinatal mental health conditions in recent years, the numbers of women experiencing PND remains unacceptably high. A history of mental health problems like depression and anxiety have been consistently reported in previous literature as predictors of PND [3, 4], however few studies have measured experiences of mental health problems prospectively, and most use retrospective data collection methods which are known to be biased [5, 6]. Evidence for screening initiatives therefore is likely to be based on cross-sectional or biased data collections that use non-representative data, indicating a pressing need for longitudinal representative data.

The impact of demographic factors on PND is uncertain with inconsistent findings reported for low income [7], relationship status [8, 9] and area of residence [10]. Similarly, investigations of the contribution of negative health behaviours around the time of pregnancy, such as high body mass index [11], smoking [12, 13], binge drinking [14, 15] and illicit drug use [16] on PND have also produced mixed results.

Stressful life events and social experiences have been associated with risk of PND such as experience of domestic violence [17] and experience of stressful life events [18, 19]. Social support has also been related to PND [20, 21] with mothers who report lower levels of social support more likely to experience PND. Measures of birth events and pregnancy health have also been investigated, again, with mixed results [22, 23]. Indeed, most analyses of the risk factors related to PND have examined events immediately prior to pregnancy and childbirth, with little published on more distal experiences of stressful life events, long-term health behaviours or impact of early life experiences.

A large-scale study by Milgrom et al [4] examined the relationship between several antenatal risk factors and PND in Australian women, taking measures of PND and risk factors during pregnancy and again six weeks postpartum. The authors reported previous mental health and lack of partner support were key predictors of PND. However this study did not take into account the cumulative impact of multiple births using a longitudinal perspective.

While previous research has highlighted a range of potential risk factors for PND, no studies to date have examined a comprehensive set of risk factors at a population-level using data collected prospectively. The aim of this longitudinal prospective study was to explore the relationship between a range of psychological, health and social factors and PND and to construct a model of the long and short-term predictors of PND. Specifically, we explored whether women who have experienced a range of psychological issues, stressful life events, have a history of poor health behaviours and who have experienced adverse reproductive and childbirth events are more likely to experience PND, taking into account a range of demographic factors.

## Methods

Data collected over a 13-year period from the 1973–78 cohort of the Australian Longitudinal Study on Women’s Health (ALSWH) was used to construct a model of the predictors of PND. ALSWH is an ongoing population-based study of the health and well-being of women in Australia, with over 40,000 participants recruited in 1996 (baseline) in three cohorts born in 1973–78, 1946–51 and 1921–26. Participants were selected via the national health insurance database (Medicare) and complete self-report mailed surveys on a three-yearly basis [24]. Women in the 1973–78 cohort were surveyed in 1996 (Survey 1), 2000 (Survey 2), 2003 (Survey 3), 2006 (Survey 4) and 2009 (Survey 5). Data from Survey 5 were used in combination with data from Surveys 1–4 in order to measure both proximal and distal predictors of PND. The research protocol was approved by University of Newcastle (H-076-0795) and University of Queensland (2004000224) Human Research Ethics Committees and all participants provided written informed consent to participate.

At Survey 5, participants completed a series of questions about the birth of each of their children including reporting the dates of birth of their children and diagnosis or treatment for PND for each child (“were you diagnosed or treated for: postnatal depression” yes/no).

For the birth of each child, several measures of pregnancy, birth and postpartum factors were reported in Survey 5 for each child. Pregnancy factors included occurrence of gestational diabetes, gestational hypertension, antenatal depression and antenatal anxiety (yes/no). Childbirth factors included emotional distress during labour, elective caesarean, emergency caesarean, preterm birth, gas, epidural, forceps, vaginal tear, episiotomy, medical removal of placenta, excess blood loss, greater than 36 hour labour, low birth weight (responses were yes/no). Postpartum factors included being diagnosed or treated for postnatal anxiety, and breastfeeding for less than six months.

Using the date of birth for each child, other risk factors were evaluated using the closest survey in time to the birth of the child (either before or after), using the date of survey return. Demographic measures included income stress, qualifications, employment status, sexual orientation and marital status, and area of residence. Social support was evaluated using the Medical Outcomes Study Social Support Index [25]. The index contains four factors–‘Emotional/informational support’, ‘Tangible support’, ‘Affectionate support’ and ‘Positive social interaction’. For the purposes of this analysis, the last two factors were collapsed into one factor following a factor analysis.

A proportional life events score was calculated using a list derived from Norbeck [26] for the twelve months prior to the survey. Physical and mental well-being was assessed with the SF-36 instrument [27], using the general health and mental health subscales. The scales are both measured on a continuum 0–100, and quartiles were developed for further analysis. Stress was evaluated using the Mean Stress scale (0–4) devised by ALSWH where a higher score indicates higher stress, and four categories were used in analysis. The Life-Orientation Test-Revised was used to evaluate optimism versus pessimism on a scale of 0–24, and categorised in to four categories. A higher category score indicates optimism. Body mass index (BMI) was calculated for each survey and a dichotomous variable measuring history of being overweight or obese (BMI>25) was subsequently created.

History of pregnancy, birth and postpartum events measures were created for each child. For example, if, during the pregnancy of any older sibling the mother experienced ‘gestational diabetes’, the history of gestational diabetes variable was coded as ‘yes’ for the index child. Prior pregnancy factors included history of gestational diabetes, gestational hypertension, antenatal depression, antenatal anxiety and previous PND. Childbirth and postpartum factors were the same as for the index birth.

History of events prior to the birth of each child was created using the date of birth of the child and the date of survey return. For example, if the mother had reported experiencing depression in 1996, the history of depression variable would be set to ‘yes’ for all children born after 1996. If the mother reported experiencing depression in 2004 for the first time, the history of depression variable for any children born prior to 2004 would be set to ‘no’ and for any children born after 2004 it would be set to ‘yes’. History of self-harm or suicidal ideation was evaluated at each survey, as was history of being in a violent relationship with a spouse or partner. For each child, the mother’s mental health history was assessed using self-report of diagnosis or treatment for mental health conditions (depression, anxiety).

At each survey, participants were asked about their experience of a range of reproductive events such as stillbirths, miscarriages, terminations and ectopic pregnancies. A history variable for each of these events was created, updated for each child to incorporate previous reproductive events for the mother. A history of poor health behaviours such as tobacco use, illicit drug use and risky alcohol use were evaluated for each woman at the time of birth for each child using the survey data collected prior to each birth. Tobacco use was evaluated at each survey using a number of questions such as ‘How often do you currently smoke cigarettes or any tobacco products?’ and ‘In your lifetime, would you have smoked at least 100 cigarettes (or equivalent)?’. These items were used to evaluate smoking status in keeping with the categories developed by the Australian Institute of Health and Welfare as ‘never smoker’, ‘ex-smoker’, ‘irregular smoker’, ‘weekly smoker’ and ‘daily smoker’[28]. A new variable for tobacco history was developed per child using the date of birth of the child and the date of survey completion. If the mother was categorised as either an ex-smoker, irregular smoker or current smoker (weekly or daily) at any survey prior to the date of birth of the child, the newly created ‘tobacco history’ variable was coded as ‘yes’. A similar procedure was used to create an illicit drug history variable. If the mother reported using any illicit drugs at a survey prior to the birth of the child, the ‘illicit drug history’ variable was set to ‘yes’. A history of risky drinking was evaluated using a similar strategy, where risky drinking is defined as consuming more than 15 drinks per week.

Missing data for most variables in this analysis was 1–2% of all responses, with the exception of social support. The most recent report of social support was used using the date of birth of the reference child and the date of survey completion. This method of evaluating social support yielded 17–19% missing data for the newly created variables due to incomplete survey waves by a number of participants. In order to maximise data, any missing data was replaced with the results of the social support items from Survey 5, as social support has been widely considered a stable trait and unlikely to have changed over the span of the surveys [29]. Analyses were conducted both with and without imputed data for the 17–19% missing and the relationship with postnatal depression was not significantly different. For all other variables, missing data was left as missing due to the large sample size and flexibility with the method of data analysis employed.

Outcomes were evaluated for each child born for each woman, resulting in stacked data (i.e. multiple rows for each mother to represent each birth). Children born to the same mother were accounted for using multi-level models. Within each row, data were collated concerning the birth of that child, history of previous birth experiences, status of the mother that time (using the nearest survey) and previous history of other predictors accumulated from previous surveys.

All analyses were conducted by CC and PF, however all authors had access to the data. Analyses were conducted in SPSS 19 [30]. The study is longitudinal in nature as it was based on the children born for each mother in the cohort, which occurred over time. The data for each mother are correlated and as such, a multi-level modelling approach was used to examine the temporal effect of children on the development of PND. Generalised linear mixed models were used to take into account number of children per mother (level 1: within mother—each child; level 2: between mothers) and using a logit link function (for binary outcome). The order of birth was examined as an influence on PND, as was the duration of time between births. A longitudinal analysis approach was used to model possible predictors and antecedents of PND by evaluating the risk factors for the time when each child was born. Potential risk factors were assessed individually prior to building the multivariate model. Those factors that are significantly associated with PND (p<0.001) were subsequently evaluated in a multivariate mixed model. Due to the large sample size and the numerous tests being conducted, a strict p-value criteria was used when evaluating the univariate results [31]. This approach minimised the risk of a multiple comparison problem where multiple tests with a larger p value may results in false positives [32]. A correlation matrix of all variables was conducted to check for multicollinearity (evaluated at .9). To account for oversampling of women from rural and remote areas, results were weighted based on area of residence at Survey 1.

## Results

Participants were aged 31–36 when they completed Survey 5 in 2009. There were 8200 participants who completed Survey 5, and of these, 5219 women (63.6%) reported ever giving birth to a child. These are the participants included in the current study, with a total of 10407 births reported. The occurrence of PND related to any child was reported by 820 (15.7%) of the mothers.

While significant, there were only weak associations found between postnatal depression and postnatal anxiety (r = 0.23, n = 9970, p = .001) and antenatal depression (r = 0.15, n = 9970, p = .001). These findings indicate that while related, these constructs are independent.

At the univariate level, several factors were not significantly associated with PND (at p>.001), and were therefore not included in the multivariate model. These variables were pregnancy, birth factors for index child and previous children (gestational diabetes, gestational hypertension, elective or emergency caesarean, preterm birth, gas, epidural, forceps, vaginal tear, episiotomy, removal of placenta, excess blood loss, >36 hour labour, low birth weight, breastfeeding for less than 6 months), history of events prior to birth (stillbirth, miscarriage, ectopic pregnancy, termination, illicit drugs, risky drinking), other recent risk factors (employment, partner status, area of residence, overweight).

All variables that were significant at the .001 level in the univariate models were evaluated in a multivariate mixed model (Table 1). Nine factors remained associated with PND in the multivariate model: history of depression, history of PND, history of postnatal anxiety, low SF-36 Mental Health Index, occurrence of postnatal anxiety, antenatal depression, antenatal anxiety, emotional distress during labour, and breastfeeding for less than 6 months. Two of these factors were negatively associated with PND: a history of postnatal anxiety and having antenatal anxiety during the pregnancy of the index child. The other seven factors were positively associated with PND. The strongest positive associations with PND were observed for postnatal anxiety (OR = 13.79, 95%CI = 10.48, 18.13) and antenatal depression (OR = 9.23, 95%CI = 6.10, 13.97). Importantly, birth order was not significantly associated with PND, and first births were no more likely than other births to be associated with PND.

**Table 1:** Results from the final multivariate model for predictor of PND, using Multivariate Adjusted Odds Ratios (OR) and 95% Confidence Intervals (95% CI).

		No PND	PND	Unadjusted OR	Adjusted OR	P value
		N (%)	N (%)	(95% CI)	(95% CI)	
***Current Pregnancy*, *birth*, *postpartum factors (index child)***						
Gest. Hypertension		843 (9.0)	143 (13.3)	1.50 (1.21, 1.85)	1.15 (0.92, 1.45)	.22
Antenatal depression		74 (0.8)	154 (14.3)	20.44 (14.21, 29.39)	9.23 (6.10, 13.96)	< .001
Antenatal anxiety		115 (1.2)	100 (9.3)	7.18 (5.04, 10.22)	0.39 (0.23, 0.67)	< .001
Emotional distress		1548 (17.2)	442 (44.4)	3.48 (3.00, 4.04)	1.77 (1.49, 2.09)	< .001
Breastfed <6mths		3854 (44.3)	562 (57.7)	1.708 (1.47, 1.96)	1.29 (1.12, 1.50)	< .001
Postnatal anxiety		177 (2.0)	296 (29.7)	24.67 (19.46, 31.27)	13.72 (10.43, 18.03)	< .001
***Other risk factors (from survey most recent to birth)***						
Social Support–Emotional / informational	None/Little	251 (3.4)	53 (6.6)	2.41 (1.72, 3.38)	1.16 (0.76, 1.76)	.50
	Some of the time	705 (9.4)	130 (16.2)	2.15 (1.70, 2.72)	1.25 (0.93, 1.68)	.15
	Most of the time	2335 (31.3)	275 (34.3)	1.47 (1.22, 1.76)	1.05 (0.86, 1.28)	.62
Social Support—Tangible	None/Little	507 (6.8)	92 (11.6)	1.88 (1.43, 2.49)	0.89 (0.64, 1.25)	.50
	Some of the time	1102 (14.9)	144 (18.1)	1.56 (1.24, 1.95)	0.97 (0.77, 1.23)	.81
	Most of the time	2361 (31.8)	248 (31.2)	1.28 (1.06, 1.54)	1.05 (0.88, 1.26)	.60
Social Support—Positive social interaction	None/Little	113 (1.5)	25 (3.1)	2.12 (1.33, 3.40)	0.78 (0.45, 1.35)	.37
	Some of the time	479 (6.4)	105 (13.1)	2.64 (2.04, 3.41)	1.07 (0.77, 1.50)	.69
	Most of the time	1722 (23.1)	224 (28.0)	1.50 (1.25, 1.80)	0.97 (0.80, 1.19)	.78
Proportion of life events	.100–1.000	2288 (25.5)	340 (34.1)	1.53 (1.26, 1.49)	1.12 (0.90, 1.38)	.32
	.060 0 .099	2238 (24.9)	251 (25.2)	1.21 (0.99, 1.87)	1.06 (0.86, 1.31)	.57
	.030–.059	2369 (26.4)	226 (22.7)	1.01 (0.82, 1.24)	1.08 (0.88, 1.34)	.46
SF36 Mental Health Index	0–51.99	1241 (13.8)	373 (37.4)	7.16 (5.51, 9.29)	2.19 (1.68, 2.86)	< .001
	52–67.99	1497 (16.7)	230 (23.1)	3.61 (2.74, 4.74)	1.48 (1.14, 1.92)	.004
	68–87.99	4153 (46.3)	319 (32.0)	1.97 (1.53, 2.54)	1.20 (0.98, 1.48)	.084
SF36 General Health	0–61.99	1539 (17.1)	310 (31.1)	3.15 (2.56, 3.86)	1.13 (0.89, 1.42)	.32
	62–71.99	1104 (12.3)	151 (15.2)	2.15 (1.70, 2.72)	1.10 (0.87, 1.39)	.43
	72–86.99	3295 (36.7)	345 (34.6)	1.65 (1.37, 1.98)	1.11 (0.93, 1.33)	.24
Income stress	Extremely stressed	601 (6.7)	129 (13.1)	2.66 (1.10, 3.54)	0.84 (0.61, 1.16)	.30
	Very stressed	1102 (12.3)	186 (18.9)	1.91 (1.48, 2.47)	0.89 (0.67, 1.18)	.42
	Mod. stressed	1977 (22.1)	240 (24.3)	1.61 (1.27, 2.03)	0.98 (0.76, 1.26)	.87
	Somewhat stressed	3590 (40.2)	300 (30.4)	1.10 (0.88, 1.38)	0.94 (0.75, 1.18)	.60
Education	Year 12 or less	2653 (30.0)	340 (34.7)	1.44 (1.21, 1.72)	1.09 (0.91, 1.30)	.37
	Non-univ. tertiary	2186 (24.7)	284 (29.0)	1.46 (1.21, 1.76)	1.17 (0.98, 1.40)	.09
Partner Status	Not partnered	8069 (90.1)	840 (84.8)	1.47 (1.21, 1.80)	1.15 (0.92, 1.44)	.22
Mean Stress	1.09–4.00	2177 (26.2)	148 (15.6)	1.39 (1.07, 1.82)	1.36 (1.06, 1.75)	.02
	.73–1.08	1747 (21.0)	215 (22.7)	2.63 (2.04, 3.40)	1.27 (1.00, 1.61)	.06
	.45–.72	2177 (26.2)	148 (15.6)	1.39 (1.07, 1.82)	0.96 (0.78, 1.20)	.74
LOT-R Optimism	13–15.99	1815 (20.2)	204 (20.5)	1.38 (1.12, 1.70)	1.05 (0.84, 1.31)	.66
	16–17.99	2051 (22.9)	198 (19.9)	1.54 (1.25, 1.90)	0.96 (0.78, 1.19)	.73
	18–24	3306 (36.8)	241 (24.2)	2.73 (2.25, 3.31)	1.10 (0.89, 1.35)	.38
***Any history of pregnancy*, *birth*, *postpartum events (prior to index birth)***						
History of antenatal depression		60 (0.6)	52 (4.9)	1.21 (0.93, 1.55	0.64 (0.34, 1.20)	.16
History of antenatal anxiety		63 (0.7)	42 (3.9)	4.10 (2.57, 6.55)	2.20 (1.18, 4.10)	.014
History of emotional distress in labour		1087 (11.6)	239 (22.2)	1.78 (1.50, 2.11)	0.94 (0.76, 1.16)	.56
History of PND		370 (4.0)	256 (23.8)	7.21 (5.90, 8.80)	5.60 (4.23, 7.40)	< .001
History of postnatal anxiety		180 (1.9)	87 (8.1)	2.51 (1.84, 3.44)	0.34 (0.21, 0.55)	< .001
***Any history of events prior to index birth***						
History of self-harm		575 (6.2)	130 (12.1)	2.05 (1.62, 2.59)	0.93 (0.72, 1.19)	.56
History of violent relationship		859 (9.2)	152 (14.1)	1.54 (1.25, 1.89)	0.96 (0.77, 1.20)	.71
History of depression		980 (10.5)	283 (26.3)	2.95 (2.47, 3.52)	1.53 (1.24, 1.88)	< .001
History of anxiety		481 (5.2)	110 (10.2)	1.98 (1.53, 2.57)	0.89 (0.65, 1.21)	0.45
History of tobacco		4379 (48.8)	541 (54.3)	1.29 (1.11, 1.50)	0.97 (0.84, 1.12)	.65

*All values were weighted by area of residence at Survey 1 to compensate for oversampling of rural and remote areas*.

## Discussion

Results from the multivariate model reinforced the notion that mental health predictors are strongly associated with PND. The strongest relationship was observed for concurrent postnatal anxiety. The co-morbid relationship between anxiety and depression is well documented [33] and these results confirm that this relationship also holds in the vulnerable postpartum period. Significant results were also found for antenatal depression, emotional distress during labour, SF36 MHI score 0–51.99, history of depression or PND. These findings confirm previous cross-sectional and short-term longitudinal studies [4, 34] that found that reported history of mental health conditions is related to PND. Surprisingly, history of postnatal anxiety was significantly related to PND however the relationship was inverse to expected (Adjusted OR = 0.33, 95%CI = 0.21, 0.54). These results may be due to women receiving treatment for postnatal anxiety, and therefore experiencing an overall reduction in emotional health deficits.

Contrary to previous findings, several key factors were not related to PND in the multivariate model. In our study, no demographic measures remained in the final model, although several other studies have found a significant relationship between demographics such as income, education, single marital status, younger age and PND [35, 36]. Social support was not significant in the final multivariate model even though a significant relationship in the univariate model was detected. These results indicate that there is no evidence to support an association between demographic and social support factors and PND once other predictors are taken into account.

A major strength of this paper was the use of data collected from a large, representative sample of childbearing women. The use of longitudinal data collected prospectively allowed us to examine more accurately the predictors of PND as retrospective data collection may be biased. In addition, the use of multiple surveys allowed recall bias to be minimised and data harmonisation to be conducted. The multivariate mixed model approach utilising a conservative inclusion critierion also indicated that the results are robust and reliable.

Due to age restrictions of this cohort of women when recruited in to ALSWH, we were not able to examine age of motherhood as a factor which has been reported as significant in cross-sectional studies. A further limitation of this study is the use of a self-report measure of PND as the outcome variable. However, a diagnostic measure would have only detected current cases of PND, whereas the self-reported diagnosis measure allowed us to collect incidences of PND per child.

In conclusion, this study is the first to assess long and short term risk factors for PND in a broadly representative sample of Australian women in a longitudinal context. Over 15% of women reported experiencing or being treated for PND, which attests to the significance of this problem. The multivariate results indicate that understanding a woman’s mental health history plays a very important role in the detection of those who are most vulnerable to PND. These findings also indicate that treatment and management of depression and anxiety earlier in life and during pregnancy may be protective of later PND. The findings of this project provide clear direction for the development of clinical guidelines regarding PND and support the premise of early intervention to target mental health problems in adolescence. By preventing first incidences of mental health problems, recurrences such as those during the perinatal period may also be prevented, which in turn will have a positive impact on mother-infant bonding, and maternal and infant outcomes.
